# Exploring Physicians’ Perceptions of Digital Health’s Impact on the Patient-Physician Relationship in the Primary Health Care Setting: Qualitative Descriptive Study

**DOI:** 10.2196/53705

**Published:** 2024-10-15

**Authors:** Kai Ping Sze, Qi Wei Fong, Jacqueline Giovanna De Roza, Eng Sing Lee, Shu Yun Tan

**Affiliations:** 1 National Healthcare Group Polyclinics Singapore Singapore; 2 Lee Kong Chian School of Medicine Nanyang Technological University Singapore Singapore

**Keywords:** patient-physician relationship, patient communication, trust, primary care medicine, digital health, primary care, longitudinal care, policy, implementation

## Abstract

**Background:**

Digital health has become essential for effective clinical practice. However, the successful adoption of digital health is dependent on the strength of the patient-physician relationship. The patient-physician relationship shapes the quality of care and impacts health care outcomes, especially in primary care. However, the impact of the increasing use of digital health on the patient-physician relationship is uncertain.

**Objective:**

This study aims to explore the types of digital health primary care physicians use and understand their impact on the patient-physician relationship from their perspective.

**Methods:**

This exploratory qualitative descriptive study used individual in-depth interviews guided by a semistructured topic guide. We purposively sampled physicians from 6 general primary care clinics in Singapore and used thematic analysis to identify emergent themes.

**Results:**

We conducted 12 interviews. We found that primary care physicians in Singapore had minimal exposure to digital health beyond the scope of institutional implementation. The three key themes that emerged were as follows: (1) evolving roles of both physicians and patients; (2) impact on trust, knowledge acquisition, and longitudinal care; and (3) adoption and use factors of digital health impacting patient-physician relationships. The adoption and use factors comprised “social and personal,” “technical and material,” and “organization and policy” factors.

**Conclusions:**

The study identified that, while primary care physicians held mostly positive views on adopting digital health in improving the patient-physician relationship, they were concerned that digital health might erode trust, hinder proper knowledge acquisition, and reduce humanistic interaction. These concerns called for a nuanced approach to ensure that digital health would not compromise the patient-physician relationship. This could be achieved by ensuring that physicians possess the necessary skills, knowledge, and positive attitude, while health care organizations would provide robust IT capabilities and support. We recommend that education be refined and government policies on digital health adoption and use be revised to align with the goal of strengthening the patient-physician relationship.

## Introduction

### Background

The emergence of digital health has become essential for effective clinical practice in primary care. According to the World Health Organization, digital health refers to the knowledge and practices associated with the development and use of digital technologies to improve health. Digital health expands the concept of eHealth to include digital consumers, with a wider range of smart devices and connected equipment. It also encompasses other uses of digital technologies for health such as the Internet of Things, artificial intelligence, big data, and robotics [1-5].

In Singapore, the adoption of digital health in health care accelerated during the COVID-19 pandemic, including telemedicine, video consultations, and a new national database that unified the public and private health care sectors through the COVID-19 Test Repository system [6]. Although both public institution polyclinics and private general practitioner (GP) practices have adopted digital health in Singapore, the former have been more uniform in their adoption of technologies such as electronic health records (EHRs), patient portals, telemedicine, and health analytics, while the latter had more variability in their use.

The term “primary care” is used synonymously with family medicine or general practice [7]. Primary care provides personal, primary, and preventive care to patient care needs and comprehensive, continuing, and coordinated care in managing the patient and his or her family. The primary care system in Singapore is characterized by a dual structure comprising public and private providers. The public sector includes 26 polyclinics offering a wide range of services such as chronic disease management, preventive care, and maternal and child health services. The private sector consists of numerous GP clinics which deliver personalized and continuous care to patients. A polyclinic may have up to 60 clinicians, whereas a GP clinic is typically run by one or a few GPs. Overall, Singapore’s primary care system emphasizes a patient-centric approach and plays a pivotal role in maintaining the population’s health, managing chronic conditions, and reducing the burden on secondary and tertiary health care facilities.

The digital health tools of particular relevance in Singapore’s primary care for public institution care includes video consultation using Zoom (Zoom Video Communications, Inc) interface, clinical decision support using corporate system software, point of care tools, shared electronic clinical data, a secured smartphone app for physicians to communicate with specialists or for patients to have one-stop access to personal medical records, and remote monitoring of blood pressure using smart versions of clinical devices.

The patient-physician relationship is crucial in delivering high-quality health care outcomes, quality indicators, and health equity [8-13]. It is widely recognized as the most potent element in medicine as it shapes the quality of care and impacts a range of health care outcomes [8], especially in primary care, where patient-centeredness is highly valued by patients [9,10], and positive patient perceptions of the consultations have been linked to improved health outcomes [11-13].

The proliferation of digital health has shifted tasks traditionally performed in health care facilities into the patient’s home. These tools allow patients to record their own biological parameters, such as smartwatches monitoring their heart rates and rhythms. The interplay of patient-physician communication in face-to-face environments and relationship factors (eg, patient trust and patient satisfaction) could exert significant effects in promoting eHealth adoption [14]. In addition, Balato et al [15] found that patient-physician communication improved in a group receiving SMS text message interventions, suggesting that digital interventions could enhance this relationship. This democratization of monitoring and care contributes to an equal level of the patient-physician relationship as argued by Meskó et al [16]. In addition, the increased reliance on digital health has promoted the transition from the traditional guidance-cooperation model of care, in which the physician makes decisions for the patient, to a mutual participation partnership model, in which the physician and patient work together to achieve the patient’s goals [17,18]. The new model bestows equal power to both parties and they are mutually interdependent.

While digital health holds great potential to revolutionize health care by providing innovative solutions to address health-related issues for both physicians and patients, successful adoption of these digital products has to be contingent upon the strength of the patient-physician relationship. A positive patient-physician relationship is crucial to ensuring satisfactory health care encounters and effective disease management in the primary care setting. Conversely, a weak patient-physician relationship can lead to increased medicolegal issues, clinician attrition, and ultimately poorer health outcomes for patients.

### This Study

While previous studies examining the impact of digital health on primary care performance and quality have already shown that digital health holds promise in improving access, efficiency, and patient empowerment [19,20], no studies have so far explored the experiences of primary care physicians on their use of digital health and its impact on the patient-physician relationship. One reason might be due to the medicalization of clinical medicine leading to the deprioritization of humanistic medicine [21]. Another reason might be the relatively recent emergence and rapid evolution of digital health [22]. With the proliferation of digital health, it is uncertain how it would affect the patient-physician relationship. Therefore, this study aimed to fill the knowledge gap by finding out about the types of digital health that primary care physicians in Singapore use and understanding the impact of digital health on the patient-physician relationship from their perspectives.

## Methods

### Study Design and Participant Selection

The qualitative descriptive research method [23] as a study design involving individual in-depth interviews was used for this study.

Recruitment and data collection were completed within an 8-month period from June 2022 to February 2023. A total of 8 months were taken as data analysis was conducted following the data collection from each interview, and there were quality considerations in selecting interviewees capable of giving meaningful responses. We purposively identified primary care physicians to include individuals of both male and female genders with different levels of primary care experience from the professional networks of the study team. These primary care physicians were selected from 6 polyclinics which served the population in the central and northern parts of Singapore. The 6 polyclinics belong to the National Healthcare Group Polyclinics institution and hence leverage similar digital technologies. They provide a wide range of primary care services, including general medical consultations, chronic disease management, maternal and child health services, immunizations, health screenings, and diagnostic services. The polyclinics receive approximately a daily total of 5000 patients for physician consultations. The inclusion criterion was to have at least 3 years of work experience in a primary care setting, as registered medical practitioners need to have at least 3 years of work experience and relevant and recognized postgraduate qualifications to qualify as a family physician in Singapore.

### Conceptual Framework

**Figure 1:**
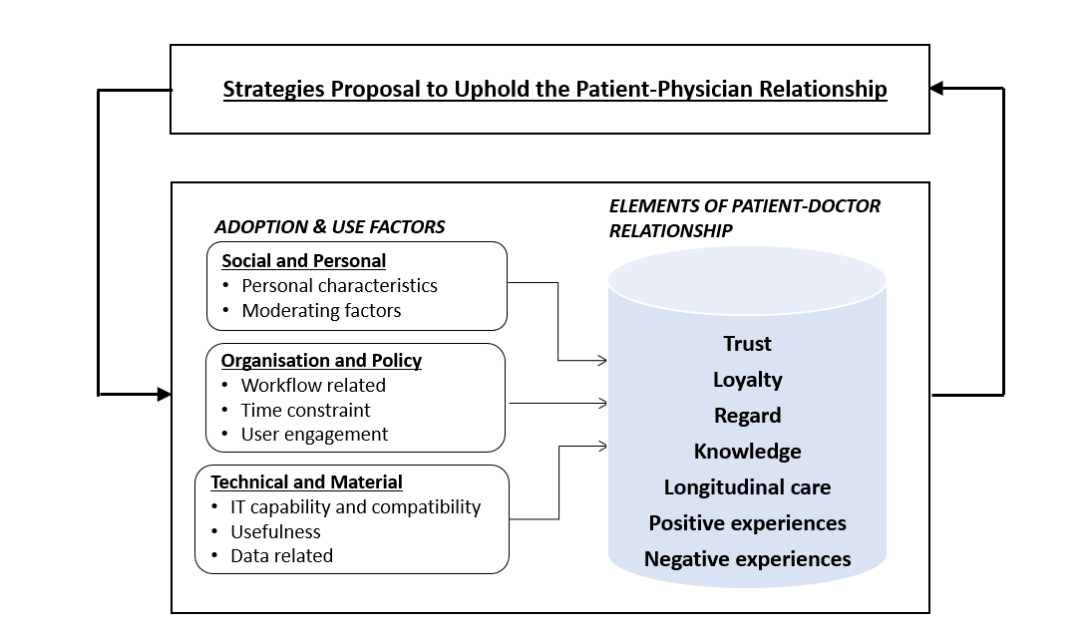
Combined conceptual framework.

The frameworks proposed by Ridd et al [24] (elements of the patient-physician relationship) and Jacob et al [25] (adoption and use factors of digital health) were used to explore the interaction of the patient-physician relationship in the context of digital health adoption and use in an iterative way. The elements in a patient-physician relationship described by Ridd et al [24] included trust, loyalty, regard, knowledge, and consultation experiences. Jacob et al [25] described the adoption and use factors as social and personal factors, technical and material factors, and organization and policy factors [25]. By combining these 2 frameworks (Figure 1), we were able to cover issues related to physician,” “patient,” “digital health,” and “trust,” as well as interdomain issues such as “patient-physician relationship,” “positive and negative experiences,” and “technology compatibility.”

### Interview Guide

We formulated a topic guide to direct the semistructured interviews (Multimedia Appendix 1) based on the conceptual framework. The topic guide comprised nondirective, open-ended questions on the following topics: intentions and experience of technological changes, present role and adoptability of digital health in primary care clinical practice, and digital health’s impact on the patient-physician relationship. The interview questions were pilot-tested and improved upon before being used. After each subsequent interview, each interviewer completed a standardized debrief with the research team and included suggested revisions to further improve the interview guide.

### Data Collection

Each in-depth interview lasted between 45 and 60 minutes and was conducted over Zoom in English and audio recorded. The interviewers were KPS and QWF, both male family physicians with approximately 8 and 15 years of clinical experience in family medicine with a master of medicine postgraduate family medicine qualification, who had no prior relationship with the participants. Another coinvestigator, either SYT (female family physician) or JGDR (advanced practice nurse), was present as an observer in every interview to capture relevant field notes and nonverbal cues. There were no other nonparticipants present. Recruitment for individual interviews was stopped upon thematic saturation. This was attained when themes and subcategories in the data became repetitive and redundant such that no new information could be gathered by further data collection [26]. No repeat interviews were required for further clarification.

### Data Analysis

Each audio-recorded interview was transcribed verbatim. Transcripts were returned to the participants for comment. Reflexive thematic analysis of the data was conducted according to the method described by Clarke and Braun [27]. Data from deidentified transcripts were analyzed alongside ongoing data collection after each interview. Coding was performed manually and data were managed using Microsoft Excel software. We began with reading and familiarization of transcripts for preanalytical understanding, followed by systematic line-by-line coding of transcripts. We independently (KPS, QWF, JGDR, and SYT) coded each transcript before clarification and agreements on the coding framework. Potential themes were then jointly developed based on the codes through consensus. Any disagreements that arose during coding and theme development were discussed among the researchers until a consensus was reached [28]. All coders met regularly to further develop the analysis and provide a check on coding consistency. Conscious attempts were made to be open to unexpected findings. The team concurred that there was no new emergence of data by the 10th interview and the point of thematic saturation [26] was reached. This was confirmed by 2 further more interviews that did not contribute to the development of new themes. Reflective memos were used throughout the data collection and analysis process to ensure the richness of data. Repeat interviews were not required.

### Ethical Considerations

Ethics approval was obtained from the National Healthcare Group Domain Specific Review Board (2021/01036), and the study was performed in accordance with the relevant guidelines and regulations laid by it. We used the COREQ (Consolidated Criteria for Reporting Qualitative Research) as our reporting framework. Written informed consent followed by sociodemographic information was obtained physically from the participants before commencing the interviews over Zoom. All participants were reimbursed with grocery store vouchers of 20 Singapore dollars (estimated US $15) as token of appreciation.

## Results

A total of 13 primary care physicians were approached and 12 (92%) of them consented to participate in the research. However, 1 (8%) declined participation over concerns with audio recording.

### Participants’ Characteristics

The participants included primary care physicians holding different designations in the public institution setting with varying years of work experience. Their ages ranged from 28 to 51 years. Their characteristics are represented in Table 1.

**Table 1:** Demographic characteristics of study participants (N=12).

Characteristic			Participants, n (%)
**Gender**			
	Men	6 (50)	
	Women	6 (50)	
**Age (y)**			
	25-34	4 (33)	
	35-44	5 (42)	
	≥45	3 (25)	
**Ethnicity**			
	Arab	1 (8)	
	Chinese	10 (83)	
	Malay	1 (8)	
**Number of years working in a primary care setting**			
	3-5	3 (25)	
	6-10	3 (25)	
	>10	6 (50)	
**Highest postgraduate training attained**			
	MBBS^a^ or MD^b^	1 (8)	
	Graduate diploma in family medicine	1 (8)	
	Master of medicine (family medicine)	8 (67)	
	Fellow of the College of Family Physicians, Singapore	2 (17)	
**Designation**			
	Family medicine resident^c^	1 (8)	
	Family physician^d^	2 (17)	
	Family physician senior staff^e^	1 (8)	
	Associate consultant^f^	6 (50)	
	Consultant^f^	1 (8)	
	Senior consultant^f^	1 (8)	

^a^Bachelor of medicine and bachelor of surgery.

^b^Doctor of medicine.

^c^Qualified physicians granted conditional or full medical registration by the Singapore Medical Council undergoing family medicine residency postgraduate training program.

^d^Registered medical practitioners with relevant and recognized postgraduate qualifications (graduate diploma in family medicine or master of medicine ([family medicine]) and with at least 3 years of relevant clinical experience.

^e^Registered medical practitioners with relevant and recognized postgraduate qualifications (graduate diploma in family medicine or membership in the Royal College of General Practitioners) and with at least 5 years of clinical experience.

^f^Registered medical practitioners with relevant and recognized postgraduate qualifications (master in family medicine, or a fellow of the College of Family Physicians, Singapore).

### Participants’ Digital Health Use Characteristics

The digital health tools that all our 12 participants used in their clinical practice were summarized in Table 2.

**Table 2:** Digital health use characteristics of study participants (N=12).

Digital health use	Participants, n (%)
Epic [29]	12 (100)
NEHR^a^	12 (100)
PTEC^b^ Home Blood Pressure Monitoring Programme [30]	12 (100)
Zoom	12 (100)
TigerText	12 (100)
HealthHub	12 (100)
Freestyle Libre capillary glucose monitoring device	4 (33)
Wearable devices	2 (17)
UpToDate smart app	1 (8)

^a^NEHR: national electronic health record.

^b^PTEC: Primary Tech-Enhanced Care.

### Emerging Themes

**Figure 2:**
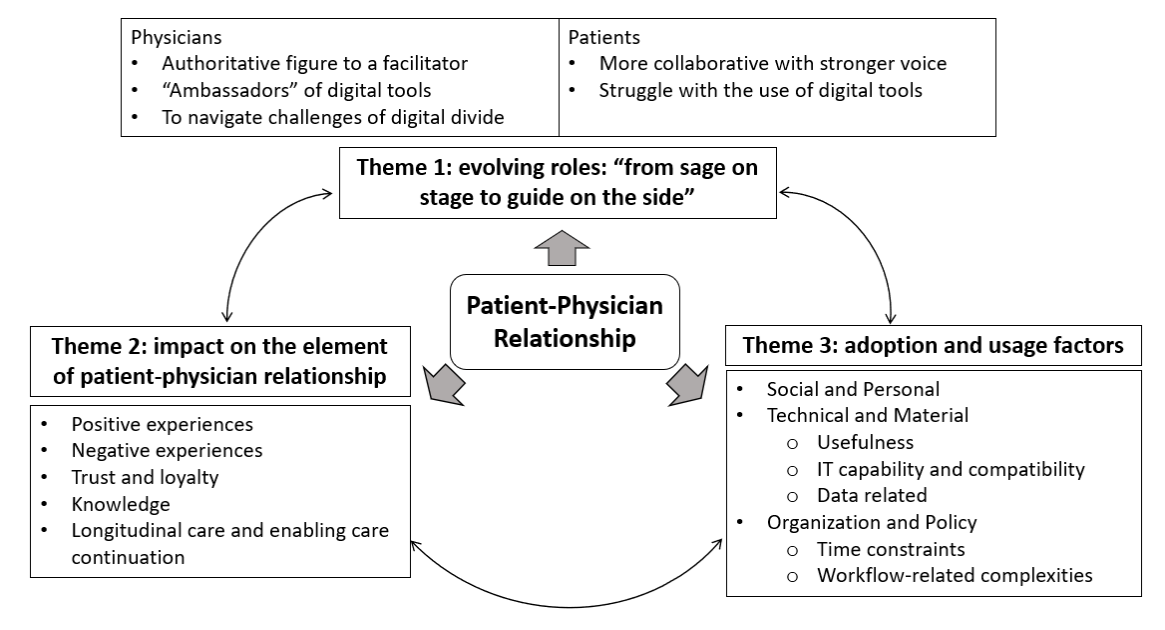
The interdependent themes and subthemes on the impact of digital tools on the patient-physician relationship from primary care physicians’ perspectives.

The results were grouped into 3 themes (Figure 2) based on the applied conceptual framework for digital health’s impact on patient-physician relationship adapted from Ridd et al [24] and Jacob et al [25].

#### Theme 1: Evolving Roles (“From Sage on Stage to Guide on the Side”)

Nowadays, patients generally prefer a more collaborative approach to a paternalistic approach, with patients playing a more active role in their own health care decisions by getting more involved in discussion and incorporating their beliefs and preferences. Primary care physicians would gather information from the patient and combine it with their own medical knowledge to develop safe and reasonable management plans. This collaborative approach allowed patients to have a more significant voice in the final plan:

In the past, the decision is very simple right? What I know is the best for you is going to be what I recommend to you, but now it is different, now I know what is best for you but you give me this other information from Google, and you said that you trust it a lot so how are we going to integrate it so that you don’t disagree.... By telling the patient the pros and cons, which is [part of] the decision-making process for the doctor, we eventually come to a conclusion with the patient’s voice being a big part of the final plan.

PC5, female family physician

The democratization of digital information reduced information asymmetry and shifted the role of physicians from an authoritative figure to being a facilitator of patient education and behavior change. As patients had preexisting knowledge, primary care physicians needed to adapt their approach and act as advocates for their patients’ health, guiding and nudging them toward desirable behaviors and correcting any misconceptions they may have. Overall, the patient-physician relationship has become more analytical and collaborative in recent years, with physicians taking on a more facilitative role in promoting patient education and behavior change:

In this digital day and age, patients would have already come in with a certain knowledge about their conditions, certain opinions, and you try and advocate and nudge them in a way that will promote desirable behaviour, [and] perhaps correct misconceptions.

PC8, male senior consultant

Primary care physicians also had to act as “ambassadors” for digital health to educate patients on their potential benefits for managing clinical conditions. By assuming an active role in promoting digital literacy and facilitating access to appropriate resources, physicians could empower patients to take a more active role in their own care. One participant introduced a national health application platform to an older patient successfully, fostering a sense of self-worth in him. By empowering patients to use digital health, the physician demonstrated respect for his patient’s capabilities and autonomy, thereby strengthening their relationship:

But because he is elderly and I introduced him to HealthHub...I think it was dignity that “Oh, I’m eighty years old, I can still use phone and HealthHub, you know.” And I taught him how to do it.

PC12, female associate consultant

One participant cautioned primary care physicians to be mindful of the health care inequality. Specifically, physicians who insisted on using certain digital health tools, such as glucometers or telemonitoring of blood pressure, might inadvertently create unintended barriers to care for patients who could not afford them. Patients might feel discriminated against being given optimal care because they did not know, could not afford, or did not have support at home, leading to a breakdown in trust:

If I keep insisting on using the glucometer or tele-BP, for safety reason, when the patient cannot afford it, patient might feel that they are not given optimum care.... They might also feel left behind because of lack of knowledge and they really cannot understand what is HealthHub, how to download and all that. Sometimes... they might feel that they are being discriminated against.

PC12, female associate consultant

Participants reported on their experiences with older patients who struggled to use digital devices. Furthermore, the study found that for digital health to be effective for older patients, these tools needed to be matched to the level of technological proficiency of the individual patient. Failure to consider these factors could lead to patients having difficulties in adoption and suboptimal use, thus affecting the quality of care and the patient-physician relationship. While the participants remained committed to promoting the use of digital health, they were mindful that some patients might face challenges due to the digital divide. Primary care physicians would therefore need to navigate such situations with tact and resourcefulness:

Of course there is the patients’ tech-savviness. It can be a very good tool, but if the patient is unable to utilise it because they don’t understand or they don’t know how to use it, then it’s a useless tool to the patient.

PC5, female family physician

Nevertheless, some patients would still expect a more prescriptive approach from their primary care physicians, which could lead to misunderstandings and a patient’s perception of incompetence on the part of the physician. To maintain a strong patient-physician relationship, physicians must strike a nuanced balance between the 2 approaches and adjust their communication style based on the needs and preferences of each patient:

I made the mistake of trying to offer too many options and was viewed as being an incompetent doctor n because the patient had expected a more paternalistic and prescriptive kind of style. On one hand, in the past, it used to be legacy effect that we are prescriptive or paternalistic, but nowadays, it has changed, so it’s a balance of both.

PC10, female associate consultant

#### Theme 2: Impact on Trust, Knowledge Acquisition, and Longitudinal Care

##### Positive Experiences

The overall impact on the participants was positive when digital health was applied to the background of an existing patient-physician relationship:

Patient-doctor relationship improves when patients’ medical conditions are well controlled [due to digital health] leading to greater satisfaction from both parties.

PC7, male senior family physician

Physicians were able to efficiently synthesize clinical information of patients and provide holistic care through a consolidated view of medical history, telemonitoring data charts, laboratory results, medications, and opinions of different care providers. For instance, EHR systems increased the visibility and the clarity of information for both physicians and patients, which in turn increased their confidence in technology. This, in turn, allowed for more effective communication between physicians and patients, as physicians could provide a more holistic picture of the patient’s health status and treatment plan:

Integrated system enhances the patient experience because now you can access the information from everywhere, you can piece the story together and come out with a better, more consolidated care plan for the patient. You can update them about things that they have done elsewhere, and that actually increases that rapport and gives you that holistic approach to the patient.

PC3, female consultant

##### Negative Experiences

However, most participants felt that clinical documentation systems were time-consuming and reduced eye contact and physical interaction with patients. Simultaneously, patients would perceive physicians as unempathetic and paying less serious attention to their complaints when the latter spent more time on the EHR system. This could upset the dynamics of the consultation when patients perceived physicians as being more interested in completing a checklist than in attending to their needs:

Doctor is not necessarily doing less for the patient but purely because more time is spent gleaning all the info from the computer, the patient might feel that the doctor is not paying as much attention to them and not taking them seriously in terms of their complaints. I think that will affect the dynamics of the consultation.

PC5, female family physician

##### Trust

Digital health had a dual effect on the trust shared between physicians and patients, which could be either beneficial or detrimental.

With telemonitoring tools, physicians could adjust medications better and improve patient involvement in their care based on shared clinical information on common platforms, such as home blood pressure and capillary glucose readings. Caregivers were also more involved in the care process and able to provide valuable feedback to the physicians, which could improve treatment outcomes. Technology has enhanced the patient-physician relationship through fostering a shared sense of responsibility for the patient’s health and promoting collaborative decision-making. This should ideally result in increased patient satisfaction and better health outcomes:

The positive thing is that with the BP machines at home, SMBG [self-monitoring blood glucose], I can better titrate my patients’ medication. Patient can also be able to be more involved in their care, as are their caregivers.... I could also show them that “Hey, actually this works, you just trust us.... we are keeping a close watch on you.”

PC12, female associate consultant

Patients who read the same web-based information as provided by their physicians were more likely to trust the information validated by their physicians and exhibit increased confidence in their physicians’ clinical expertise. There was also increased partnership when both physicians and patients had equal access to clinical knowledge and data, which could help to build a patient-physician relationship based on trust, communication, and accountability:

Patients are more knowledgeable so whatever you tell them, they are more convinced because they are also reading the same thing on the internet. So in that way that helps a little bit with their confidence there when delivering this kind of information.

PC7, male senior family physician

However, most (9/12, 75%) study participants expressed concerns that the misuse of internet could lead to patient distrust, conflicts with clinicians, and deterioration of the patient-physician relationship. Digital health could contribute to misinformation when patients use search engines and social media to research their health issues without distinguishing reliable from unreliable sources. Physicians might worry that patients trust web-based information more than the advice they receive from them. This could undermine trust and lead to a breakdown in the patient-physician relationship. Patients who relied heavily on digital health to self-diagnose or research their health issues might feel hesitant to discuss their concerns with their physician, leading to reduced communications with their primary care physicians. Similarly, physicians struggled to determine what their patients were reading and where they obtained their sources of information:

Things like google can be double edged sword. There’s a lot of information out there, [but if] the patient has no insight on the right information then it can lead to a lot more detriment down the road. By then it will create a disparate or a distrust with the clinician.

PC3, female consultant

Unfamiliar technology initiated by patients also created a sense of uncertainty and unease among physicians. The quote mentioned subsequently also underscored the importance of physicians being proficient in the use of digital health when recommending them to patients. If they were not knowledgeable with the tools, it could undermine patient trust and confidence in their care and hence negatively impact the patient-physician relationship:

If you ask the patient to adopt this digital tool and you don’t know how to use the tool, it reflects quite badly on you as a physician because it doesn’t instil confidence in the patient and I would hate to say that it even causes the patient to mistrust your advice because you just don’t seem to know your stuff.

PC5, female family physician

Establishing trust in a new relationship and maintaining trust in an existing patient-physician relationship using digital health could be challenging. One participant highlighted a potential challenge faced by primary care physicians in building trust with newer patients, particularly when digital health tools such as teleconsultation were used as part of the care process. One potential explanation for this phenomenon is that the use of teleconsultation might interfere with the interpersonal aspects of care that are critical to building trust and rapport between patients and providers. Physicians might also be perceived as more distant or impersonal when communicating through digital channels, which could create barriers to developing a meaningful relationship with patients. Another reason could be that patients might be more skeptical of health care providers who relied heavily on digital health, particularly if they perceived these tools as substitutes for traditional forms of care:

For newer patients when I’m still trying to build that relationship, I do find that sometimes it does either cause it to take a bit longer or it can become a little bit uh I wouldn’t really call it contrived, but generally they will take a little longer time to trust me...

PC3, female consultant

If I say in a two-year timeframe, I’ve seen you physically once, and I’ve done six video consults over time, do I think that trust will be eroded? I think to some extent it will be, because there’s only that much we can do over Zoom.

PC8, male senior consultant

##### Using Digital Health to Acquire Knowledge

Digital health was also considered convenient for both patients and physicians. It provided fast access to clinical questions when needed so that physicians would have the means to quickly find clinical answers. Paradoxically, primary care physicians did not experience a reduction in patients’ trust when they searched for medical information on the web. Instead, they might feel a lack of trust from patients if they are not as proficient as their patients in using digital health for medical information. In other words, physicians were expected to have extensive medical knowledge, but patients also expected them to be adept in digital resources to enhance their diagnoses and treatment plans:

When having a question that someone asks which you don’t have the answer immediately, having a tool in your hands to search for the answers quickly will be helpful.

PC1, male associate consultant

The need for physicians to be more familiar with these technologies than their patients highlighted the importance of providing patients with clear instructions and support in using them, which could further strengthen the patient-physician relationship:

You need to know the tool better than your patients in order to use it as a physician, right, so I think that is one major issue as well.

PC5, female family physician

Most participants (10/12, 83%) agreed that the rise of the internet has made primary care physicians’ jobs more challenging. Some contended that patients, nowadays, challenge physicians’ information with the evidence they find on the internet. Patients often came to their appointments with strong convictions based on compelling information they had read or watched on the web, which made it difficult for physicians to persuade them to change their minds. Consequently, physicians often needed to find creative ways to persuade patients and communicate differently by getting more objective facts and trustworthy sources to explain to patients. Physicians would also need to fight against misinformation and synthesize the information patients find on the internet to give the best advice:

With the current advent of the internet and the availability of info out there, there is a lot more misinformation that we have to fight... Our role now becomes more than just first level information providers, we are now someone who needs to integrate whatever info that they find on the internet with what we know and then give our best practice advice.

PC5, female family physician

I think in this modern age we have seen so many times when patients bring up their emails or their articles and say, “see doctor, cholesterol medicine is harmful for my body.” You gotta think of more creative ways to counter those arguments, to convince them to take their medicine. I don’t contest the fact that they have found something. I will find a different way to persuade them.

PC4, male family physician

##### Longitudinal Care and Enabling Care Continuation

One participant highlighted that a strong patient-physician relationship and increased interaction were crucial for improving patient outcomes. With the use of digital health, these interactions could occur more often and effectively, leading to improved patient engagements and better long-term health outcomes. Effective communication between physicians and patients was important in addressing patient concerns picked up via digital health:

It can be potentially better if there are more touchpoint with the healthcare provider, or there’s greater degree of interactions.

PC2, male family physician

Digital health has revolutionized care continuation and augmented patient care beyond the physical boundaries of the clinic through virtual consultation and increased touchpoints:

If I see a patient in my clinic and then I say, “Ok we do a Zoom call the next time round... It provides you more convenience, [and] we’re able to monitor you outside the confines of the clinic setting.” So there is an advantage where technology can augment patient care.

PC8, male senior consultant

However, increased use of digital health might disrupt traditional patient care-seeking behavior as patients become overly reliant on these technologies and fail to seek care when necessary:

Patients may then assume that a lot of things can be done on their own, when they still need to seek care at certain points. [That] sometimes destroys the fine line between too much doctoring vs not being able to intervene because the patient is too reliant on the digital and doesn’t want to come back when they need to.

PC3, female consultant

#### Theme 3: Adoption and Use Factors Impacting the Patient-Physician Relationship

##### Social and Personal

The participants’ prior experiences with pen-and-paper documentation and subsequent shift to EHR systems exemplified the challenges of adapting to new technology in the primary health care landscape. The transition to new digital health was described as a steep learning curve by some participants that initially impeded their ability to effectively use the tools until familiarity was achieved. Nevertheless, almost all of the participants (11/12, 92%) displayed motivation and positive attitude to overcome the steep learning curve and to avoid being obsolete, and once they attained competency in digital health’s mastery, they were able to reach out to their patients to help them overcome their technophobia:

During the initial period, there’s a steep learning curve. It will impede us at the start and only after familiar usage, then you will be able to gain, [and] reap the actual benefits of using these tools.

PC11, female associate consultant

There’s a lot tech phobia among especially all the older generations. They are very fearful how to use this, they are not very sure or not confident to use or whether they can even use it.

PC7, male senior family physician

Furthermore, 1 participant emphasized the importance of physicians’ familiarity with digital health tools in promoting their use in patient care. Primary care physicians needed to grasp a good understanding of the digital health they used to effectively communicate their benefits to patients and integrate them into their health care process:

[U]nless you are familiar with the tools that you are using, sometimes it is difficult to advocate a tool like HealthHub because you don’t use it yourself.

PC1, male associate consultant

##### Technical and Material

###### IT Capability and Compatibility

Some participants (3/12, 25%) expressed difficulty in finding solutions and technical support as a potential negative impact of using digital products. An example given by a participant was waiting for IT support for 2 months after being denied access to national electronics health records. This emphasized the potential impact on the patient-physician relationship, as delayed access to patient information could lead to delayed diagnosis and treatment, reduced patient satisfaction, and possibly a breakdown of trust. The use of digital health could enhance patient care and convenience, but it would be crucial to ensure that technical issues were addressed promptly to avoid potential negative consequences on the patient-physician relationship:

In NEHR, I don’t have access to sensitive information now, previously I did but now I don’t and I don’t know why and I’m still waiting for the IT [support] to help me out for the past 2 months.

PC1, male associate consultant

Nevertheless, an integrated health record system enabled access to more patient information, which facilitated the delivery of holistic care. Patients would be assured that their physicians were invested in their care and that ample time was devoted to discussing the clinical details. Overall, an integrated system could contribute significantly to improving the quality of care and patient satisfaction, thereby strengthening the patient-physician relationship:

When using the [Epic] system...I can visually show the patients how they are doing and, show them the charts. It also gives them that same visual reinforcement of how they are doing. It’s also very clear to them that I know what’s happening. I think that helps to reinforce the trust not just in them but in the technology that we are both investing the time in. For that, I think it has improved the patient-doctor relationship and the consultation.

PC3, female consultant

###### Data Related

Digital health records helped to keep consultation sessions fruitful by enabling the seamless sharing of information among health care providers involved in a patient’s care. This would help primary care physicians understand a patient’s medical history and ongoing treatments, leading to more convenient and personalized care and increased patient satisfaction:

When your patient doesn’t know what happened to himself or herself at first discharge and didn’t bring their memo, then at least digital records will keep the consultation fruitful.

PC5, female family physician

The ability to countercheck patient-reported medical records using electronic systems could enhance the patient-physician relationship. By ensuring the accuracy and reliability of the data, patients would feel confident that their physicians had the most up-to-date information. If discrepancies or inaccuracies were identified, physicians could engage in discussions with patients to clarify the missing details, that would promote shared decision-making and stronger bonding between them:

If the patient was not clear about his or her medical records or for some reason was not accurate in reporting, there is always the counter check you can do in the electronic system.

PC5, female family physician

However, the risk of data breach in sharing patient information through digital systems posed a threat to patient confidentiality and privacy. This could lead to a negative impact on the patient-physician relationship, as patients might feel that their personal information was not being handled with adequate care and might question the security of their medical records. A high-profile data breach incident in the past had raised awareness of the potential risks associated with sharing patient information through digital systems [31]:

If there’s a data breach, then patient confidentiality and patient private personal data is lost. So that’s the negative thing.

PC12, female associate consultant

##### Organization and Policy

###### Time Constraints

Lack of time was often a common limiting factor quoted by many participants. While the integration of digital health in health care could potentially benefit patients, it also required additional time and effort to educate patients on their use. It might take more time initially to explain the use of digital health to patients. However, once patients were comfortable using the tools, it could improve the patient experience and facilitate remote care:

Initially, it might take up more time to tell the patient about Primary Tech Enhanced Care [PTEC] Home Blood Pressure Monitoring Programme, about video consult, to teach them how to use them.

PC11, female associate consultant

Our study participants had concerns about the number of clicks and screens slowing down the process of seeing patients and increasing the time required for documentation. This could potentially lead to physicians missing important information and causing harm to patients. Technical issues and difficulty in navigating the tools also hindered patient care and could also create a sense of frustration and burnout among physicians, which would further strain the patient-physician relationship:

Epic has a lot more buttons to click to see one patient. It will definitely slow down the overall process of seeing patients.

PC5, female family physician

Sometimes if the tools are a bit cumbersome, like they take a long time to log in, or carry many technical errors.

PC2, male family physician

###### Workflow-Related Complexities

Some of the primary care physicians interviewed struggled with the complexities in workflow, including navigating complex computer systems and troubleshooting technical issues. Physicians needed to allocate more time and effort toward managing these technical aspects of health care, which might impact the quality of their interactions with patients:

20 years ago, all you do is just write S.O.A.P [Subjective, Objective, Assessment and Plan], prescription and send off. Nowadays, you have more things to do. You have to navigate through the Epic system more, and you need to know where to click. Of course, a lot of times the computer system also got issues. Disk crash, system hang, printer cannot print, so that increases the complexity compared to like 20 years ago.

PC7, male senior family physician

## Discussion

### Principal Findings

This qualitative study explored the types of digital health used in primary care and its impact on the patient-physician relationship in Singapore’s primary care with results based on primary care physicians’ perspectives. Although digital health encompassed a broad scope, our participants’ exposure to it was limited to mostly what was offered by their institution in their everyday work, with few venturing to explore other technologies such as capillary glucose monitoring systems and wearable devices.

By using applied conceptual frameworks, we linked key elements of the patient-physician relationship to the adoption and use factors of digital health. We found that primary care physicians held mostly positive views on adopting digital health in improving the patient-physician relationship. These included views that patients would become more empowered, and better holistic care could be provided as the roles of physicians and patients evolved with time.

Despite the positive impact of digital health, the physicians interviewed still viewed it as a double-edged sword in the patient-physician relationship, because digital health undermined the relationship through misinformation, decreased humanistic interactions, and aggravated the digital divide. While an integrated health system could provide holistic care, the use of digital health also requires a capable IT system and implicit skills, knowledge, and positive attitude from physicians to navigate the workflow complexities and reach out to their patients within the consultation time constraints.

Finally, some study participants emphasized the potential impact of the digital divide on patients. Older patients, who are most in need of health care services, might end up losing out in the digital race. With the aging population, health inequalities and inequities in access to health care, digital health, and health outcomes area likely increase [32].

### Comparison With Prior Work

The findings resonated with a web-based survey [33], which found a global consensus among primary care physicians on the role of digital health in driving greater patient empowerment. Most of the participants started adopting the bio-psycho-socio-digital paradigm approach to patient care, as they highlighted the attitudes, skills, and knowledge necessary for this approach. The findings aligned with those of Győrffy et al [34], who indicated that modern technology and the spread of digital information had accelerated the shift from a paternalistic model of the patient-physician relationship to a more collaborative and cooperative model. In this new model, digitally proficient physicians view themselves as guides who take on protective and informational roles, adeptly managing the description, collection, and dissemination of reliable content on the web [34]. This perspective is echoed by health care professionals in the study by Macdonald et al [35], who embraced the idea of patients as “partners” to help improve outcomes by educating themselves and conscientiously monitoring their condition and behavior [35]. In addition, a survey [36] found that Singapore had the second-highest percentage of physicians who believed digital health had a positive impact on the quality of treatment decisions and patients’ health outcomes; our study participants generally viewed that digital health could provide better holistic care.

As the adage went, “there is only one cardinal rule: one must always listen to the patient” [37]. Similarly, our findings reminded primary care physicians to listen to their patients to be cognizant of their care preferences so that they could act as guides for patients, particularly in navigating through the digital health landscape. Our findings mirrored the findings of a recent mixed methods study [38] that identified preparing with intention, listening intently and completely, and exploring emotional cues as 3 of the 5 practices that have the potential to enhance the patient-physician relationship during a clinic encounter. In addition, doctors must prioritize their primary goal as treating patients, with technology as adjuncts. By adopting a bio-psycho-socio-digital paradigm shifts in patient engagement and emphasizing on developing the soft skills of patient communication and the technical skills of digital health in primary care education, a n ideal patient-physician relationship in this digital age could be preserved.

Both our study and the study by Kludacz-Alessandri et al [39] emphasized patient satisfaction as a crucial outcome of teleconsultations. Our findings further substantiated that an integrated health records system enhances patient satisfaction by enabling more holistic and personalized care, mirroring the convenience highlighted in the study by Kludacz-Alessandri et al [39]. In addition, both studies acknowledged challenges with digital tools. Our study identified issues such as, the complexity of digital systems and potential data breaches, while the study by Kludacz-Alessandri et al [39] underscored the digital divide and technical difficulties as barriers to effective teleconsultations.

Our research also uncovered an intriguing “tension” that suggested a disparity in how older and younger adults perceived the reliability of physicians and the internet as sources of health information. According to Low et al [40], older adults only seemed to trust health care professionals for health information, while the internet was used to supplement existing health knowledge or for general health information. In contrast, most of the physicians (8/12, 67%) we interviewed expressed that they felt undermined by patients’ increasing trust in internet for information and the need to reconvince them of evidence-based treatment.

### Implications for Practice

First, physicians could consider tailoring their approach to a patient’s level of digital health literacy, as well as being comfortable and competent in enhancing the relationship with digital tools that patients are familiar with. Second, exposure to digital health and the importance of relational ethics [41] during medical education could improve the patient-physician interaction in the bio-psycho-socio-digital paradigm [42]. Third, robust IT capabilities and support should be provided by health care organizations to ensure that physicians leverage digital tools effectively. Finally, government policy might be needed to address challenges and ensure equity in access to digital health [43], and community partnerships and health coaching could reduce costs and help enhance the patient-physician relationships. The fragile and evolving patient-physician relationship needs to be incubated by a broader meso- or macro-organizational, cultural, and geopolitical environment. Supportive actions such as extending consultation time and maximizing enablers to uplift digitally disadvantaged patients could strengthen the patient-physician relationship.

### Implications for Research

The digital divide has emerged as a significant social determinant of health. Despite high internet and mobile device penetrations in countries such as Singapore [44], possessing such technology does not guarantee that users have the skills or motivation to use them. Therefore, accessing and engaging with digital primary care becomes a complex and multifactorial process. Given the complexity, our completed research analysis provides a comprehensive understanding of the impact of using digital health among a clinically diverse group of patients in primary care on patient-physician relationship building, with a focus on how the digital divide affects the relationship. Our next study will further explore qualitative data to capture patient perspectives in greater depth.

### Strengths and Limitations

The major strength of our work is the novelty of this study, conducted in Singapore and exploring physicians’ perspectives on digital health’s impact on the patient-physician relationship in primary care services. In addition, we used a conceptual framework to provide a theoretical basis for the study.

Nevertheless, this study also has limitations. The qualitative research was exploratory in nature and the accounts from the participants were within the unique context of a public health care institution in Singapore’s health care system, and thus, we advise caution about transferring our findings to other health care systems or local GPs from the private practice. Although purposive sampling was done, all participants interviewed were exposed to similar types of digital health implemented by their institution, with none of them discussing technologies such as artificial intelligence, big data, and robotics.

### Conclusions

This study identified that all the primary care physicians interviewed had largely similar exposure to digital health and held mostly positive views on adopting digital health in improving patient-physician relationship, as it improved patient empowerment and collaborative care among health care professionals and aided clinical decision-making processes. However, they were also concerned that digital health might erode trust between physicians and patients, hinder proper knowledge acquisition, and reduce humanistic interactions that are essential for building a strong rapport. These concerns indicated a need for a nuanced approach to ensure digital health does not compromise the patient-physician relationship.

To fully use digital health in enhancing the patient-physician relationship, physicians should possess skills, knowledge, and a positive attitude to navigate the workflow complexities and connect with their patients. This should be supported with robust IT capabilities to leverage digital tools effectively. As primary care physicians continue to adapt to the full potential of digital health, we recommended that primary care education be refined and government policies on digital health adoption and use be revised to align with the goal of strengthening the patient-physician relationship.

## Appendix

Multimedia Appendix 1 Topic guide for interview.

## Abbreviations

COREQ: Consolidated Criteria for Reporting Qualitative Research
EHR: electronic health record
GP: general practitioner

## Data Availability

The datasets analyzed during the study are available from the corresponding author on reasonable request.
