# Supplementary material for: Exploring Physicians’ Perceptions of Digital Health’s Impact on the Patient-Physician Relationship in the Primary Health Care Setting: Qualitative Descriptive Study
Source: J Med Internet Res. 2024 Oct 15;26:e53705. doi: 10.2196/53705 (PMC11522646; doi:10.2196/53705)
Supplement: Multimedia Appendix 1 [file jmir_v26i1e53705_app1.docx]

**Supplementary Guide**

| Topic guide |  |  |
| --- | --- | --- |
| Study Information | | |
| 1. Study title | Exploring doctors’ perceptions of digital health’s impact on the patient-doctor relationship in Singapore’s primary healthcare setting | |
| 1. Date of interview |  | |
| 1. Interviewee serial number |  | |
| 1. Sex | Male / Female | |
| 1. Ethnicity | Chinese / Malay / Indian / Others: ___ | |
| 1. Age |  |  |
| 1. Highest education level attained | Bachelor’s degree / Postgraduate diploma / Master’s degree / Fellowship in Family Medicine / Doctorate | |
| 1. Years of practice experience |  |  |
| 1. Work position | FM Resident trainee / Resident Physician / Family Physician / P Senior Staff / Associate Consultant / Consultant / Senior Consultant | |
| 1. Location of practice |  |  |
| 1. Digital health used before    1. Tele-visits | Tele-consultation / Video-consultation | |
| - 1. Clinical decision support tools | Epic / Best practice advisory / Drug-drug interaction warning / Care gaps | |
| - 1. Point of care / Work enhancement tools | TigerText application / Freestyle Libre / PTEC-HT | |
| Section A: Intentions and experience of technological changes | | |
| Discussion item   1. What are the technological changes that you experienced throughout your clinical practice? 2. A) What are some of the types and examples of “digital health in healthcare” that you know of?   B). What are the current digital health that you use in your consultations? | Prompt  Electronic health records, smartphone clinical applications, EPIC, Zoom for video-consults, remote monitoring, wearables, HealthHub etc | Concepts  Calibrate definition of digital health according to WHO definition |
| Section B: Present role and adoptability of digital health in primary care clinical practice | | |
| 1. How do you keep yourself updated with these digital health |  | Adoption and usage factors |
| 1. What motivates you to adopt digital tolls in your clinical practice? | Better patient care? Cost savings? | Adoption and usage factors |
| 1. Do you actively engage your patients with digital health? Which are the ones and why? |  | Adoption and usage factors |
| 1. What are the barriers that you face when adopting them in clinical practice? | Increased cognitive load? Physician burnout? Lack of integration? | Adoption and usage factors |
| Section C: Digital health and patient-doctor relationship | | |
| 1. How would you describe a “patient-doctor relationship”? |  | Calibrate definition of patient-doctor relationship |
| 1. What are your guiding principles in building a patient-doctor relationship? | Being familiar with patient; Well-prepared consult; Provide a listening ear; Connect with patients; Explore emotional cues | Calibrate definition of patient-doctor relationship Elements of patient-doctor relationship |
| 1. Do you think that technology influences the dynamics of the patient-doctor relationship? | In terms of physician-patient concordance? Trust in physician? Patient enablement? | Impact on elements of patient-doctor relationship |
| 1. Have digital health influenced the communication patterns between doctors and patients in a consult? | Advent of internet? More time spent explaining decisions? Shared decision making? | Evolving roles |
| 1. A) How else have digital health positively impacted our patient-doctor relationship? 2. B) How else have digital health negative impacted our patient-doctor relationship? | Patient compliance influencing inter-personal relationship with doctor? Decision making process affected? | Impact on elements of patient-doctor relationship |
| 1. Do patients who are more tech-savvy get a different standard of care? How so and why so? |  | Evolving roles Digital divide |
| 1. Has the information asymmetry in digital health in the current digital era changed your role as a doctor and how so? |  | Evolving roles |
